# Nutrition-induced remission of type 2 diabetes: mechanisms, clinical evidence, and future directions-a mini review

**DOI:** 10.3389/fcdhc.2026.1792614

**Published:** 2026-02-20

**Authors:** Denisa Pescari, Simina Mihuta, Andreea Bena, Roxana Pui, Corina Paul, Dana Stoian

**Affiliations:** 1 Department of Doctoral Studies, “Victor Babeș” University of Medicine and Pharmacy, Timișoara, Romania; 2 Department of Advanced Ultrasound, DRD Medical Center, Timișoara, Romania; 3 Center for Molecular Research in Nephrology and Vascular Disease, “Victor Babeș” University of Medicine and Pharmacy, Timișoara, Romania; 4 Department of Pediatrics, “Victor Babeș” University of Medicine and Pharmacy, Timișoara, Romania; 5 Discipline of Endocrinology, Second Department of Internal Medicine, Victor Babeș University of Medicine and Pharmacy, Timișoara, Romania

**Keywords:** diabetes, diets, metabolism, nutrition, obesity

## Abstract

Type 2 diabetes mellitus (T2DM) is considered a chronic, progressive and irreversible condition; however, evidence accumulated over the past decade demonstrates that remission of this disease can be achieved through targeted nutritional interventions. This mini review aims to synthesize current data regarding nutritional interventions that may lead to T2DM remission, with emphasis on the underlying mechanisms, clinical outcomes and implications for both research and clinical practice. Evidence supporting the role of structured dietary strategies, including low-energy diets, the Mediterranean dietary pattern, ketogenic approaches, and time-restricted eating, in improving glycemic control and facilitating remission is analyzed, mainly through the reduction of ectopic fat and the improvement of insulin sensitivity. Remission is more likely in individuals with a short duration of diabetes, a more favorable initial glycemic status, and significant and sustained weight loss, particularly when visceral and hepatic fat are reduced. Beyond weight loss, emerging data suggest that meal timing, macronutrient quality, and adherence to nutritional interventions play important modulatory roles. Despite promising results, current evidence is limited by heterogeneity in remission definitions, short follow-up periods, and difficulties related to implementation in real-world clinical practice and long-term sustainability. Nevertheless, there are insufficient data regarding predictors of relapse and the safety of nutritional interventions in vulnerable populations. In the future, research should prioritize long-term randomized studies primarily oriented toward remission and using personalized nutritional interventions. Therefore, nutrition-induced T2DM remission represents a feasible and clinically relevant therapeutic objective, with the potential to redefine current management strategies under the application of individualized, multidisciplinary, and long-term recommendations.

## 1 Introduction

Type 2 diabetes mellitus (T2DM) is a multifactorial metabolic disease resulting from the interaction between genetic predisposition and environmental exposures, with its global prevalence closely mirroring the rise in obesity worldwide (1). The onset of T2DM is strongly associated with chronic positive energy balance and excess adiposity, initiating a complex pathophysiological process involving insulin resistance, impaired incretin signaling, low-grade systemic inflammation, and altered metabolic intermediates (2). Emerging evidence suggests that gut-derived metabolites and microbiota-host interactions may further modulate insulin sensitivity and glucose homeostasis in susceptible individuals (3).

A central mechanistic feature of T2DM is ectopic fat accumulation in insulin-sensitive organs, particularly the liver, pancreas, and skeletal muscle, leading to organ-specific metabolic dysfunction (4). Metabolic dysfunction-associated steatotic liver disease (MASLD) contributes directly to increased hepatic glucose output, while intrapancreatic fat accumulation impairs β-cell function and first-phase insulin secretion, resulting in progressive hyperglycemia (5). These metabolic abnormalities frequently coexist with hypertension and dyslipidemia, reflecting shared underlying insulin-resistant pathways (6). Traditionally, T2DM has been regarded as a chronic and progressive disease requiring lifelong pharmacological management (7). Despite modern glucose-lowering therapies, a substantial proportion of patients develop microvascular and macrovascular complications that significantly reduce life expectancy and quality of life (8). Current projections estimate that, in the absence of effective preventive strategies, the number of individuals living with T2DM will reach over 600 million globally by 2045 (9).

Weight loss represents the most effective non-pharmacological intervention for improving insulin sensitivity and reducing cardiometabolic risk in individuals with T2DM (4). Studies employing intensive dietary interventions or bariatric surgery have demonstrated rapid improvements in hepatic and skeletal muscle insulin sensitivity, often preceding significant changes in overall body weight (10). These early metabolic benefits are closely associated with reductions in ectopic fat stores rather than generalized fat mass alone (11). The concept that T2DM remission is achievable through sustained weight loss has been robustly demonstrated in randomized controlled trials (12). In the Diabetes Remission Clinical Trial (DiRECT), maintenance of weight loss exceeding 10 kg resulted in diabetes remission in a substantial proportion of participants at two-year follow-up (13). Imaging studies from mechanistic sub-analyses indicate that remission is accompanied by normalization of pancreatic fat content and partial restoration of β-cell function, supporting reversibility of disease pathophysiology (14).

Despite growing evidence supporting nutrition-induced remission, clinical guidelines provide limited prescriptive guidance regarding optimal dietary composition (15). Both the American Diabetes Association and European Association for the Study of Diabetes emphasize individualized dietary approaches while acknowledging that multiple dietary patterns may be effective when they achieve sustained negative energy balance (16). This lack of specificity has contributed to heterogeneous clinical practice and conflicting interpretations of dietary evidence (17). Adherence to any energy-restricted dietary intervention can induce weight loss, regardless of macronutrient distribution, provided that compensatory increases in energy intake do not occur (18). However, inter-individual variability in adherence and metabolic response often confounds direct comparisons between dietary strategies in clinical trials (19). Moreover, poorly designed restrictive diets may predispose to micronutrient deficiencies or adverse metabolic effects if not adequately supervised (20). Long-term weight loss maintenance remains a critical determinant of sustained metabolic benefit and diabetes remission (21). Evidence suggests that greater initial weight loss predicts improved long-term maintenance, highlighting the importance of effective early interventions combined with structured behavioral support (22). Behavioral and lifestyle programs play a pivotal role in sustaining dietary adherence and preventing weight regain over time (23).

Remission of T2DM is currently defined as achievement of glycemic indices below diagnostic thresholds in the absence of glucose-lowering medication, most commonly HbA1c <48 mmol/mol (6.5%) or fasting plasma glucose <7.0 mmol/L (24). Variability in remission definitions and duration of medication withdrawal across trials complicates direct comparison of outcomes (25). Nonetheless, converging evidence indicates that nutritional interventions can induce remission primarily through sustained weight loss and reduction of ectopic fat burden (14).

Low-energy (LED) and very-low-energy (VLED) diets have demonstrated the strongest evidence for inducing remission, particularly in early T2DM (13). Low-carbohydrate (LCD) and ketogenic diets (KD) improve glycemic control and reduce medication use, although remission appears largely dependent on achieved weight loss rather than carbohydrate restriction alone (19). High-protein diets may support weight loss maintenance through enhanced satiety and thermogenesis, though long-term remission data remain limited (26). Mediterranean dietary patterns consistently improve cardiometabolic risk factors and may facilitate remission when associated with sustained weight reduction (27). Time-restricted eating has emerged as a promising strategy for improving insulin sensitivity, although evidence for durable diabetes remission remains preliminary (28).

The aim of this mini-review is to critically evaluate the mechanisms, clinical evidence, and future directions of nutrition-induced remission of type 2 diabetes, with particular emphasis on sustainable dietary strategies capable of reversing disease pathophysiology.

## 2 Pathophysiological rationale for dietary-induced remission

The pathophysiological basis for dietary-induced remission of T2DM lies in the reversibility of key metabolic abnormalities driven primarily by chronic energy excess and ectopic fat accumulation (4). Excess caloric intake relative to individual adipose tissue storage capacity promotes lipid spillover into non-adipose organs, particularly the liver and pancreas, where intracellular lipid accumulation disrupts insulin signaling and β-cell function (29). Hepatic steatosis increases hepatic glucose production through impaired insulin-mediated suppression of gluconeogenesis, contributing to fasting hyperglycemia early in the disease course (30).

In parallel, accumulation of pancreatic fat has been shown to impair β-cell glucose responsiveness and first-phase insulin secretion, a hallmark defect in T2DM progression (5). Mechanistic studies demonstrate that reduction of intra-organ fat content through substantial energy restriction leads to rapid restoration of hepatic insulin sensitivity, followed by gradual recovery of β-cell function, supporting the concept that these defects are not irreversible (10). This sequence of metabolic normalization has been described as the “twin-cycle hypothesis,” whereby chronic energy excess drives self-reinforcing cycles of liver and pancreas fat accumulation that can be interrupted by sustained negative energy balance (31).

Dietary interventions capable of inducing significant weight loss reduce circulating free fatty acids and improve adipose tissue insulin sensitivity, thereby decreasing lipid flux to ectopic sites (32). The resulting improvement in whole-body insulin sensitivity is accompanied by reductions in inflammatory mediators and lipotoxic intermediates that contribute to insulin resistance and β-cell dysfunction (33). Low-grade systemic inflammation, a characteristic feature of T2DM, is attenuated following dietary-induced weight loss, further enhancing insulin signaling pathways in peripheral tissues (34).

Beyond weight loss per se, dietary composition may modulate metabolic pathways relevant to remission, including insulin secretion dynamics, hepatic glucose metabolism, and metabolic flexibility (35). However, evidence suggests that improvements in glycemic control and remission are primarily mediated by the magnitude and durability of energy deficit rather than by specific macronutrient manipulation alone (17, 35, 36). This observation is supported by studies demonstrating comparable metabolic benefits across diverse dietary patterns when equivalent weight loss is achieved (17).

Importantly, early intervention appears critical, as shorter diabetes duration is associated with greater β-cell functional reserve and higher likelihood of remission following dietary intervention (13). This temporal relationship underscores the progressive nature of β-cell failure in T2DM and highlights the therapeutic window during which metabolic abnormalities remain reversible (14). Collectively, these findings provide a robust pathophysiological rationale for dietary-induced remission of T2DM, positioning sustained negative energy balance and reduction of ectopic fat as central mechanisms underpinning disease reversal.

The primary cardiovascular benefit of weight management through lifestyle and behavioral interventions lies mainly in the prevention of metabolic diseases and cardiovascular risk. Conversely, once cardiometabolic pathology is established, the effectiveness of these interventions may be limited (33). Evidence indicates that remission of type 2 diabetes mellitus through such lifestyle and nutritional approaches depends both on the rapidity of weight loss and on its magnitude, particularly through the reduction of adipose tissue mass (34). Even a moderate weight loss (up to 10%) can improve β-cell function and insulin sensitivity by reducing pancreatic and hepatic fat, with reversal of glucolipotoxicity contributing to the restoration of first-phase insulin secretion and metabolic flexibility (35). In addition, hypocaloric diets reduce pro-inflammatory cytokines involved in insulin resistance, such as TNFα and IL-6 (36). Therefore, behavioral and lifestyle interventions can induce remission of type 2 diabetes mellitus.

## 3 Evidence for major nutritional interventions leading to type 2 diabetes remission

### 3.1 Low energy diet and very low energy diet

LED is defined by the provision of a daily caloric intake between 800 and 1200 kcal; however, less restrictive caloric ranges also exist (800–1800 kcal/day) (36, 37). The recommendations for this category of diet therapy relate to its role in promoting weight loss over relatively short time intervals and, implicitly, its effectiveness on insulin resistance (38, 39). VLED are characterized by an even more restrictive caloric intake, below 800 kcal/day and above 400 kcal/day (36). In this type of diet, predominantly liquid and semi-solid foods are consumed, with the aim of achieving significant weight reduction, particularly in individuals with a BMI over 40 kg/m², over short time periods of up to 12 weeks (28). Adequate medical supervision is required to prevent reductions in muscle mass secondary to insufficient protein intake, as well as to prevent other metabolic complications or even death (40).

Nevertheless, it has been observed that significant caloric restriction leads to substantial weight loss, with long-term success (41), and over longer periods, greater reductions have been recorded compared with LED (37). Furthermore, the DIADEM-I study included 147 participants under 50 years of age who were diagnosed with type 2 diabetes mellitus. These individuals underwent a hypocaloric diet with an intake between 800 and 820 kcal/day over a period of 12 weeks. Dietary reintroduction after this period led to a significant impact among the subjects. Consequently, independent management of caloric restriction was identified (37). At the same time, another objective related to physical activity was achieved, namely reaching 10,000 steps per day, respectively 15 minutes over 7 days. These factors led, over the course of one year, to remission in 61% of individuals with type 2 diabetes mellitus, as a result of a 12% weight loss (37).

The DiRECT study and its extension demonstrated that remission of type 2 diabetes mellitus can be achieved through an intensive dietary intervention in patients with a short duration of disease (12). The protocol included a very low-calorie diet based on total diet replacement (≈825–853 kcal/day) for 12 weeks, followed by gradual food reintroduction and weight maintenance support. Remission rates were 46% at 1 year, 40% at 2 years, and declined to 10% at 5 years, being strongly correlated with the magnitude of weight loss, particularly in patients who achieved a reduction of >10% of initial body weight (38).

The Look AHEAD (Action for Health for Diabetes) study is the largest multicenter randomized trial that evaluated the effects of an intensive lifestyle intervention in adults with type 2 diabetes mellitus (39). An observational analysis of the cohort included 5.145 overweight participants, aged 45 to 76 years, with a mean diabetes duration of approximately 5 years, randomized either to an intensive weight loss intervention or to standard care with diabetes education. The intervention combined caloric restriction (1.200–1.800 kcal/day), reduction of saturated fat intake, and an increase in physical activity to ≥175 minutes/week. Compared with the control group, the intensive intervention was associated with significantly higher rates of sustained remission at 2 years (9.2% vs. 1.7%) and at 4 years (3.5% vs. 0.5%), with remission occurring more frequently among participants who achieved substantial weight loss.

A recent randomized controlled trial evaluated the impact of intermittent caloric restriction on remission of type 2 diabetes mellitus. The study included 72 patients, with a mean age of approximately 53 years and a mean disease duration of up to 11 years. The intervention consisted of cycles of severe hypocaloric dieting (840 kcal/day for 15 days), alternated with periods of ad libitum feeding, within the framework of adherence to national dietary guidelines for diabetes. At the end of the intervention, remission was observed in 44.4% of participants in the intervention group, associated with a mean weight loss of approximately 9%. These data suggest that intensive dietary interventions can induce remission of T2DM, particularly when initiated early, before the development of advanced pancreatic β-cell dysfunction (40).

### 3.2 Low carbohydrates and ketogenic diet

The introduction of weight loss as a mandatory criterion in the definition of remission proposed by the Association of British Clinical Diabetologists and the Primary Care Diabetes Society (ABCD/PCDS) may limit the recognition of euglycemia achieved through low-carbohydrate or ketogenic dietary interventions in situations where weight loss is minimal or absent (42). In such cases, normalization of glycemic control may be attained exclusively through carbohydrate restriction, even in the absence of antidiabetic pharmacological therapy. Consequently, euglycemia induced by low-carbohydrate or ketogenic diets without significant weight loss remains insufficiently conceptualized, although this phenomenon is consistent with historical clinical observations suggesting that improvement in glycemic control is not always dependent on reductions in body mass (41). For example, Laura R. Saslow et al. demonstrated that individuals with type 2 diabetes mellitus randomized to a very low-carbohydrate ketogenic diet, integrated into an online lifestyle program, achieved superior glycemic control and greater weight loss compared with those who followed an online program based on a conventional low-fat diet. These findings suggest that digital delivery of low-carbohydrate/ketogenic interventions may facilitate large-scale implementation of effective self-management strategies for T2DM (43).

The growing interest in low-carbohydrate diets has led to a substantial expansion of research examining their effects on metabolic health (44, 45). However, there is no consensus regarding the definition of carbohydrate intake that characterizes these diets (46). According to the Acceptable Macronutrient Distribution Range (AMDR), carbohydrates should provide 45–65% of total energy intake; therefore, diets falling below this threshold are frequently classified as low-carbohydrate (45). Other classifications apply more restrictive thresholds or criteria based on absolute intake, defining these diets by a consumption below 200 g/day or, in non-ketogenic variants, between 50 and 150 g/day (47–49). Nevertheless, epidemiological data suggest that a moderate carbohydrate intake, approximately 50–55% of total energy, is associated with a lower risk of mortality, indicating that the type and quality of carbohydrates may have greater metabolic relevance than the total quantity consumed (45, 49).

In contrast to low-carbohydrate diets, the KD is characterized by a high fat intake and an extremely low carbohydrate content (50). KD aims to induce ketosis, defined as a metabolic state in which the body primarily utilizes lipids as the main source of energy rather than glucose, as occurs in a balanced diet (51). As a result of this metabolic adaptation, a substantial reduction in adipose tissue occurs, with a concomitant decrease in insulin resistance, alongside preservation of lean tissue secondary to the protective effects of ketone bodies (52). At the same time, this nutritional approach has been shown to reduce total body weight, with the primary focus on adipose tissue mass rather than muscle mass (53). Conversely, it has been observed that this dietary pattern may limit muscle mass development over time due to a significantly reduced carbohydrate intake, given the important role of carbohydrates in muscle glycogen replenishment (54). Currently, research suggests that KD may make an important contribution to the remission of type 2 diabetes mellitus by normalizing glycemic control and reducing or, in some cases, even discontinuing antidiabetic therapy (36, 55, 56). For example, interventional studies have shown that severe carbohydrate restriction, which is characteristic of a ketogenic diet, leads to rapid and sustained percentage reductions in HbA1c, partially independent of weight loss, through reductions in glycemic levels and insulin requirements (57, 58). Furthermore, a systematic review and meta-analysis reported that ketogenic diets are associated with higher remission rates at 6 months compared with control diets, although long-term sustainability remains variable (36).

### 3.3 High-protein diet

Nutritional interventions with a high protein intake are considered a potential supportive strategy for improving glycemic control and, implicitly, for the remission of T2DM, because of their proven effects on weight loss, particularly through preservation of muscle mass, effects on satiety, and improvement of insulin sensitivity (26, 59–61). It has been observed that an increase in protein intake leads to enhanced thermogenesis and satiety, with positive effects on spontaneous caloric reduction and, ultimately, sustained body weight reduction, which represents the strongest predictor of type 2 diabetes mellitus remission (62). Furthermore, high-protein diets have demonstrated the ability to reduce postprandial glycemic peaks secondary to delayed gastric emptying, as well as through incretin-mediated mechanisms, particularly GLP-1 and GIP, which stimulate insulin secretion, thereby demonstrating improved short-term glycemic control (26, 63). At the same time, favorable outcomes regarding glycemic control have been reported in individuals with type 2 diabetes mellitus following a high-protein diet, with significant reductions in HbA1c values compared with traditional diets based primarily on carbohydrate intake, independent of weight loss (26, 64).

In the assessment of cardiovascular risk among individuals with diabetes mellitus (which is considered very high based on this diagnosis alone), factors beyond the total quantity of protein consumed are important, with apparently contradictory data reported (65, 66). Although there is substantial evidence supporting the benefits of high-protein diets associated with reduced total caloric intake and diabetes risk, these effects differ according to protein source (67, 68). In the pan-European EPIC-InterAct study, higher total protein intake, particularly from animal sources, was associated with a higher incidence of type 2 diabetes mellitus after adjustment for relevant risk factors, whereas plant-derived protein was not correlated with increased risk (69). These findings highlight the importance of both protein quantity and quality, both of which play an essential role in the evaluation of cardiometabolic risk in patients with type 2 diabetes mellitus.

Preservation of skeletal muscle mass within high-protein regimens may further contribute to improved peripheral glucose utilization and insulin sensitivity, particularly in individuals with obesity-related sarcopenia or long-standing insulin resistance (70). Although landmark remission trials such as DiRECT primarily emphasized total energy restriction, accumulated evidence suggests that high-protein dietary patterns may facilitate remission by enhancing adherence, minimizing loss of fat-free mass, and improving metabolic flexibility during weight loss interventions (6, 12).

Protein is a key macronutrient for body composition, with intakes above the RDA (0.8 g/kg/day) associated with improved lean mass preservation (71–73). In physically active individuals, higher intakes (1.2–2.0 g/kg/day) are commonly recommended (74). When combined with moderate energy restriction (500–750 kcal/day), protein intakes exceeding 22% of total energy improve insulin sensitivity and attenuate loss of fat-free mass during weight loss in both women and men, although effects on adipose tissue mass are less well defined (75, 76).

Nevertheless, concerns regarding long-term renal safety and potential cardiovascular effects require careful patient selection and individualized protein targets, highlighting the need for long-term studies specifically designed to evaluate high-protein diets as a strategy for achieving durable remission of type 2 diabetes mellitus (75, 77).

### 3.4 Mediterranean diet

The Mediterranean diet is the dietary pattern known for its high intake of minimally processed foods, plant-based foods, cereals, fruits, vegetables, legumes, nuts, olive oil (the main dietary source of fat), moderate consumption of fish, and minimal consumption of processed red meat. This dietary model is also characterized by a consistent reduction in the risk of developing type 2 diabetes mellitus (T2DM) (78). Compared with other categories of diet therapy (standard diabetes diets, calorie-restricted diets, or low-fat diets), superior adherence to the Mediterranean pattern has been shown to lead to significant improvements in glycemic control in individuals with T2DM (79, 80). Furthermore, alongside improvements in HbA1c and fasting plasma glucose values, an amelioration of cardiovascular risk has been observed among these individuals, classifying the Mediterranean diet as a sustainable dietary pattern for these patients (79, 80). Over the long term, it has been shown to improve insulin sensitivity through reductions in BMI, as well as to decrease the need for glucose-lowering medication, thus remaining a dietary strategy with high efficacy and a superior remission rate of T2DM (81).

There are several mechanisms that demonstrate the beneficial effects of this dietary pattern: reduction of oxidative stress secondary to the attenuation of chronic low-grade inflammation, as well as improvement of endothelial function and prevention of atheromatous plaque formation (82, 83). Remission of type 2 diabetes mellitus can be achieved through a Mediterranean nutritional approach, particularly in individuals with recently diagnosed T2DM (27, 81, 84). The effects leading to T2DM remission can be explained as follows: through percentage reduction in body weight, which increases insulin sensitivity in individuals with and without diabetes mellitus, as well as through reduction of serum levels of advanced glycation end-products (AGEs) in individuals with T2DM and associated coronary heart disease (84–87).

The potential of the Mediterranean diet to facilitate remission of type 2 diabetes mellitus extends beyond the effects of weight loss and involves specific mechanisms that include improvement of hepatic insulin sensitivity through reductions in *de novo* lipogenesis and hepatic glucose production, as well as optimization of insulin signaling at the cellular level through increased intake of unsaturated fatty acids, particularly oleic acid (88, 89). Furthermore, the Mediterranean diet favorably modulates gut microbiota composition by increasing the abundance of short-chain fatty acid–producing bacterial taxa, which have been implicated in enhanced insulin sensitivity, augmented incretin secretion, and attenuation of systemic inflammation (90). In a 12-week randomized controlled trial including adults with prediabetes and type 2 diabetes, a low-carbohydrate Mediterranean dietary pattern demonstrated a more favorable metabolic profile when compared with a well-formulated ketogenic diet (91).

Accordingly, international diabetes guidelines consistently recommend the Mediterranean diet as a first-line dietary pattern for individuals with type 2 diabetes, owing to its well-documented benefits on glycemic regulation and cardiovascular risk reduction, as well as its emerging role within structured nutritional strategies designed to induce and sustain diabetes remission in carefully selected patient populations (92).

### 3.5 Time-restricted eating

Time-restricted eating (TRE) has gained substantial attention in recent years for its capacity to induce weight loss and exert metabolic benefits (93, 94). Unlike conventional dietary interventions that prioritize caloric quantity, TRE is defined by the timing of food intake (95). Within the spectrum of fasting-based dietary patterns, such as zero-calorie alternate-day fasting, modified alternate-day fasting, and the 5:2 diet, TRE is currently the most prevalent approach (95–98). This strategy involves restricting food consumption to a daily window of approximately 4–10 hours, without systematic calorie counting, followed by fasting during the remaining hours of the day (93). In individuals with obesity, TRE has been associated with spontaneous reductions in daily energy intake of up to 550 kcal and body weight losses ranging from 5% to 10% (98–101). Importantly, the observed weight reduction appears to be driven predominantly by decreases in adipose tissue rather than lean mass (93), with clinically significant weight loss also accompanied by reductions in abdominal and visceral fat depots (101, 102).

It has been observed that TRE may indirectly support remission of T2DM through multiple metabolic pathways: synchronization of food intake with the circadian rhythm and, independent of intentional intake restriction, through improvement of metabolic regulation (103). The implementation of an early TRE dietary pattern has been shown to increase insulin sensitivity, β-cell responsiveness, and glycemic control, even in the absence of weight loss, mechanisms directly relevant to diabetes remission (28). Likewise, among individuals with prediabetes and insulin resistance, TRE has been shown to be associated with attenuation of postprandial glycemic peaks and a reduction in basal insulinemia (104). Although direct data on remission remain limited, consistent improvements in remission-related markers, such as HbA1c, hepatic insulin sensitivity, and visceral adiposity, suggest that TRE may facilitate nutrition-induced remission when integrated into structured lifestyle interventions (105). However, long-term randomized trials are currently required, given the limited evidence regarding this dietary pattern and remission of T2DM (106).

Although multiple dietary strategies have been investigated for T2DM remission, direct comparisons between approaches remain limited. As summarized in **Table 1**, dietary interventions differ substantially with respect to remission rates, long-term sustainability, safety considerations, cardiovascular effects, and target populations. This heterogeneity highlights the need for individualized dietary selection based on metabolic phenotype, comorbidities, and patient preferences.

**Table 1:** Comparative analysis of dietary strategies for T2DM remission and metabolic improvement (36, 43, 51, 53, 107).

Dietary strategy	Remission rate (short–medium term)		Long-term sustainability	Safety profile	Cardiovascular impact	Most suitable population
VLED	High (40-60% in early T2DM)	Low-moderate		Risk of micronutrient deficiencies, gallstones; requires medical supervision	Improves cardiometabolic risk short term; long-term data limited	Early T2DM, severe obesity, high motivation, close clinical monitoring
LCD/KD	Moderate-high	Moderate		Potential micronutrient deficits; transient dyslipidemia in some individuals	Generally neutral to favorable when well-formulated	Insulin-resistant phenotype, central obesity, patients preferring carbohydrate restriction
Mediterranean Diet	Low-moderate	High		Excellent safety profile	Consistently reduces CV risk and inflammation	Broad population, older adults, high CV risk
Time-Restricted Eating (TRE)	Low-moderate	Moderate		Generally safe; risk of hypoglycemia in treated patients	Modest cardiometabolic benefits	Mild T2DM, prediabetes, individuals seeking lifestyle-based approaches
Low-Fat Diet	Low	Moderate		Low risk	Modest CV benefit; limited remission data	Patients with strong fat-restriction preference
Plant-Based Diet	Low-moderate	Moderate-high		Risk of vitamin B12, iron deficiency if unbalanced	Favorable lipid and inflammatory profile	Motivated individuals, ethical/environmental preference

## 4 Determinants of successful remission

Successful remission of T2DM is primarily determined by both metabolic and clinical factors, including disease duration, baseline glycemic status, and residual β-cell function (13, 14). Available evidence indicates that shorter diabetes duration and lower initial HbA1c are associated with higher remission rates, reflecting less advanced β-cell dysfunction (14, 108). Regardless of the nutritional intervention used, the importance of weight loss, particularly with respect to the reduction of visceral, hepatic, and pancreatic fat, constitutes a key determinant of T2DM remission (4, 14). At the same time, long-term maintenance of remission is significantly influenced by a range of factors (the intensity of lifestyle optimization, adherence to the implemented diet therapy, as well as behavioral modifications) and is supported by these rather than by the nutritional intervention itself (109). Furthermore, interindividual variability in the severity of insulin resistance, adipose tissue distribution and genetic background may influence the degree of remission, supporting the importance of implementing personalized strategies to achieve remission among individuals with T2DM (110).

## 5 Clinical implications for healthcare providers

From a clinical perspective, the induction of remission through various nutritional interventions tailored to the individualized needs of patients with T2DM supports the necessity for a paradigm shift in the management of this condition, requiring the establishment of a therapeutic objective that extends beyond conventional glycemic targets, such as fasting plasma glucose and HbA1c, toward the achievement of remission. Health care professionals should prioritize the early identification of patients eligible for remission-oriented strategies, particularly those with a short duration of disease, relatively preserved β-cell function, and excess adipose tissue. Nutritional interventions should be individualized, feasible, and sustainable, considering patient preferences, associated complications, and underlying metabolic phenotype. Elements such as regular monitoring, structured behavioral support, and interdisciplinary collaboration represent integral components for optimizing long-term effectiveness and adherence. Importantly, remission of T2DM should not be regarded as a cure, but rather as a dynamic and potentially reversible state that requires ongoing monitoring and the sustained implementation of nutritional and lifestyle interventions within routine clinical practice.

## 6 Limitations of current evidence and gaps in knowledge

Although an increasing volume of important evidence in the literature supports nutrition-induced remission of T2DM, several limitations restrict the interpretation of these results. A substantial proportion of studies are characterized by short follow-up periods of nutritional interventions, less precise definitions of T2DM remission, and considerable variability in medical nutrition therapy protocols. Moreover, due to the complexity of highly structured programs used in clinical trials, their implementation in real-world clinical practice among patients with T2DM, as well as the inconsistency of long-term monitoring over an indefinite period, may represent factors that influence long-term remission. At the same time, predictors of relapse and the safety of the applied nutritional interventions must be considered in vulnerable populations, such as older adults, individuals with long-standing T2DM, or those with multiple associated comorbidities. A major limitation of the available evidence is the heterogeneity in the definition of type 2 diabetes remission across guidelines and clinical studies. As summarized in **Table 2**, remission criteria differ substantially with respect to HbA1c cut-off values, required duration of normal values of glycemia, and the necessity of complete withdrawal of glucose-lowering medication. This variability limits direct comparison between studies and may partly explain discrepancies in reported remission rates.

**Table 2:** Variability in the definition of T2DM remission across guidelines and research studies (12, 13, 23, 25, 39, 111–113).

Source/guideline	HbA1c criterion	Fasting plasma glucose	Duration of glycemic control	Requirement for antidiabetic medication withdrawal	Notes/implications
ADA Consensus (2009)	< 6.0% (partial) < 5.7% (complete)	< 126 mg/dL	≥ 1 year	Yes	Introduced difference between partial and complete remission; limited adoption in later studies
ADA–EASD Consensus Report (2021)	< 6.5%	Not mandatory	≥ 3 months	Yes (no glucose-lowering therapy)	Currently the most widely accepted definition; pragmatic for clinical and research use
DiRECT Trial	< 6.5%	—	≥ 2 months	Yes	Shorter duration criteria: may overestimate remission rates compared to stricter definitions
DIADEM-I Trial	< 6.5%	—	≥ 3 months	Yes	Applied in early T2DM; emphasizes disease duration as a key modifier
Look AHEAD Study	< 6.5%	—	≥ 1 year	Partial (some analyses allowed metformin)	Heterogeneous definitions across follow-up analyses
Observational Cohort Studies	Variable (5.7–6.5%)	Variable	Variable (3–12 months)	Inconsistent	Major source of heterogeneity and limited comparability between studies

## 7 Conclusions and future directions

In conclusion, nutrition-induced remission of T2DM represents a clinically meaningful and achievable therapeutic goal for selected patients, with increasing evidence supporting the role of structured dietary interventions as first-line strategies. Future research should prioritize long-term, remission-focused randomized trials using standardized definitions and clinically applicable protocols. The development of a personalized and sustainable approach applicable to everyone with T2DM is necessary and essential to transform remission into an integrated component of routine clinical practice, with the potential to rethink the management of patients with T2DM.
